# A Candidate EEG Spectral Index of Internally Oriented Attention: An Exploratory Comparison of Prayer and Relaxation

**DOI:** 10.3390/brainsci16030311

**Published:** 2026-03-14

**Authors:** Cristian Manea, Corina Colareza, Dana Rad, Mușata-Dacia Bocoș, Teofil Panc, Mona Bădoi-Hammami, Gheorghe Mihai Bănariu

**Affiliations:** 1 Faculty of Psychology, Titu Maiorescu University, 040051 Bucharest, Romania; cristian.manea@prof.utm.ro (C.M.); teofil.panc@prof.utm.ro (T.P.); 2 Faculty of Educational Sciences, Communication and International Relations, Titu Maiorescu University, 040051 Bucharest, Romania; 3 Centre of Research Development and Innovation in Psychology, Faculty of Educational Sciences, Aurel Vlaicu University of Arad, 310032 Arad, Romania; 4 Faculty of Psychology and Educational Sciences, Babes-Bolyai University, 400029 Cluj-Napoca, Romania; musata.bocos@ubbcluj.ro; 5 Teacher Training Department, Faculty of Psychology and Educational Sciences, Ovidius University of Constanta, 900527 Constanta, Romania; hammami.badoi@gmail.com; 6 Faculty of Medicine, Carol Davila University of Medicine and Pharmacy, 020021 Bucharest, Romania; banariu.gheorghe@gmail.com

**Keywords:** internally oriented attention, candidate transcendence index, prayer, relaxation, EEG, theta, alpha, beta, self-referential processing

## Abstract

**Highlights:**

**What are the main findings?**

- A candidate EEG-derived Transcendence Index (TI), computed as the relative balance of theta–alpha versus beta activity, was systematically associated with oscillatory patterns consistent with internally oriented attention and reflective self-processing (strongest and most stable effects found in theta–alpha; beta tended to be lower).
- Prayer and Relaxation (eyes-closed) showed descriptively distinct oscillatory patterns, suggesting that prayer engages internal-focus processes that may not be fully captured by relaxation alone.

**What are the implications of the main findings?**

- TI offers a reproducible, computation-light EEG spectral index that can be derived from portable/low-density recordings and may complement self-report measures by providing an objective proxy of internal-orientation dynamics.
- Treating Prayer and Relaxation as interchangeable internal-focus conditions may be methodologically misleading; comparative designs should model them as distinct states when studying internally oriented neurodynamics and value-laden reflection.

**Abstract:**

Background: Self-transcendence has been described in psychological literature as an orientation toward meaning beyond the individual self. However, because the present study does not directly measure transcendence as a psychological construct, we approach it cautiously as a candidate form of internally oriented attention, operationalized through EEG spectral dynamics. Although this construct has been linked to self-referential cognition and large-scale brain systems supporting internal mentation, electrophysiological evidence remains limited, especially in designs that compare spiritually oriented practices with non-spiritual internal-focus controls. Objective: We examined whether a candidate EEG-derived Transcendence Index (TI) is associated with EEG oscillatory activity across canonical frequency bands and whether prayer and relaxation show descriptively distinct oscillatory patterns. Methods: In a within-subject design, participants completed a psychological assessment battery including personality and anxiety measures and underwent EEG recording during two eyes-closed conditions (Prayer vs. Relaxation). Spectral power features were extracted for delta, theta, alpha (low/high), beta (low/high), and gamma (low/high, where signal quality permitted). We examined associations between TI and band-limited activity and explored condition-related oscillatory patterns across Prayer and Relaxation. Given the modest sample size (*N* = 39), the study was designed and interpreted as exploratory research. Results: Higher TI was associated with an oscillatory profile consistent with internally oriented attention and reflective self-processing, with the most consistent patterns observed in theta–alpha dynamics (and comparatively lower beta contribution). In addition, Prayer and Relaxation showed descriptively distinct oscillatory patterns, suggesting that prayer engages internal-focus processes that may not be fully captured by relaxation alone. Conclusions: These findings support the feasibility of examining internally oriented attentional dynamics potentially related to “transcendence” as a candidate construct through scalp EEG spectral activity. Integrating theory-informed indices with EEG features may help refine psychophysiological models of self-transcendence and inform digitally supported assessment approaches, pending further construct validation. These findings should therefore be interpreted as exploratory preliminary evidence supporting the feasibility of EEG-based indices of internally oriented attention.

## 1. Introduction

In a period marked by rapid social, technological, and cultural change, understanding universal values has become increasingly relevant for psychology, sociology, and cognitive science. Universal values are typically defined as broad axiological principles that guide action beyond situational demands and inform value-laden cognition, evaluation, and internally oriented reflection relevant to value orientation [1,2,3,4,5]. Within Schwartz’s theory of basic human values, values are conceptualized as trans-situational motivational goals organized in a circular structure that captures compatibilities and conflicts among value dimensions (e.g., universalism, benevolence, conformity, self-direction). This framework is widely used to explain how value priorities relate to behavior across domains and cultures [4]. Recent cross-national evidence further suggests that value orientations reflect stable societal and psychological patterns associated with cohesion, tolerance, and adaptive functioning [6].

Beyond descriptive mapping, values are increasingly viewed as embedded within personality organization and self-regulatory systems, influencing how individuals evaluate competing moral demands and act under axiological tension [1,2,5]. Moral neuroscience offers converging support for this view: moral judgment is shaped by interactions between affective and executive processes, supported by prefrontal circuitry and limbic systems, as demonstrated in classic moral dilemma paradigms [7]. At the psychophysiological level, interindividual variability in moral decision-making has been associated with electrophysiological markers (e.g., alpha power, delta/theta phase coherence), suggesting that value-laden cognition may be expressed in measurable oscillatory dynamics [8].

Within this broader landscape, self-transcendence has been conceptualized as a higher-order orientation that moves beyond immediate self-interest toward existential meaning. In the present study, however, the construct is treated cautiously as a candidate theoretical interpretation of internally oriented attentional states, rather than as a directly measured psychological trait. Critically, transcendence is not reducible to formal religiosity; it also encompasses the capacity to relate to universal values such as truth, justice, compassion, and responsibility toward others [9,10,11]. In personality frameworks, self-transcendence has been modeled as a distinct dimension associated with psychological well-being, altruism, and moral behavior, motivating operational measures intended to approximate axiological predispositions [12]. Complementing trait perspectives, experimental work indicates that inducing a self-transcendent mindset can modulate neural responses to persuasive health messages and predict downstream behavior change, reinforcing links between transcendence, valuation processes, and prosocial decision-making [13]. Importantly, the present study does not test behavioral outcomes; rather, it focuses on EEG-based spectral associations in a comparative prayer–relaxation design.

Advances in cognitive neuroscience have further clarified the neural systems supporting reflective and value-related cognition, implicating large-scale networks such as the default mode network (DMN), fronto-parietal control networks, and limbic circuitry in meaning-making, self-related cognition, and moral evaluation [7,14,15]. The Default Mode Network (DMN) has frequently been associated with internally oriented cognitive processes such as self-referential thought, autobiographical memory, and mind-wandering. However, the present study does not directly measure DMN activity, as scalp EEG lacks the spatial resolution necessary to identify large-scale functional networks with precision. Instead, DMN theory is used here as a heuristic framework to contextualize internally oriented attentional states that may manifest in EEG spectral dynamics [14,15]. Converging evidence suggests that experiences characterized by reduced self-focus (e.g., awe) may involve decreased DMN activity, aligning with conceptualizations of transcendence that emphasize self-decentering and expanded moral concern [16]. These network-level accounts also converge with valuation-based models highlighting medial prefrontal regions (including ventromedial prefrontal cortex) as key substrates for self-related valuation and meaning attribution [17,18].

Within this neurocognitive framing, electroencephalography (EEG) offers an efficient approach to capturing the temporal dynamics of neural activity associated with internal attention, reflective monitoring, and value-laden evaluation, given its high temporal resolution and non-invasive nature [19]. Oscillatory activity in the theta and alpha bands has been repeatedly linked to internally oriented attention, self-related processing, cognitive–affective integration, and self-regulation, making these bands particularly relevant to transcendence-relevant functioning [20,21]. Syntheses of contemplative states further suggest that inward-focused practices can modulate theta/alpha dynamics in ways that reflect shifts in attentional style and internal monitoring [20,21]. More recent evidence has associated theta activity with self-transcendent experiences and with reduced maladaptive tendencies in behavioral and affective regulation, supporting a plausible mechanistic role of theta-linked processes in transcendence-relevant functioning [22]. Comparative work indicates that prayer and secular relaxation/meditation can yield distinguishable EEG profiles, particularly in alpha and theta bands, suggesting that spiritually oriented internal focus is not necessarily interchangeable with non-spiritual relaxation at the neurophysiological level [23].

Against this background, the present study examines whether an EEG-derived candidate Transcendence Index (TI)—interpreted conservatively as a spectral proxy of internally oriented attention—is associated with EEG oscillatory activity and whether Prayer and Relaxation differentiate such oscillatory profiles within a comparative design. Specifically, we aim to (a) identify oscillatory correlates associated with higher TI values, (b) operationalize TI as a theory-informed EEG-derived proxy of internal orientation using spectral features, and (c) examine associations between TI and activity across theta, alpha, beta, and gamma frequency bands. This approach aligns with emerging directions in digital psychometrics and digital phenotyping, which seek to complement self-report instruments with physiological and behavioral signals to increase measurement objectivity and improve ecological relevance [24]. Specifically, the proposed TI was formulated as a ratio between theta–alpha activity and beta activity in order to capture the relative predominance of internally oriented reflective processing over externally activated analytic processing. Theta and alpha were combined because they were treated as complementary rather than redundant oscillatory components: theta was assumed to reflect internal monitoring and reflective control, whereas alpha was assumed to index sensory gating and stabilization of internally generated mentation, especially under eyes-closed conditions. Beta activity was used as the reference term because it is more commonly associated with alert, task-oriented, and externally engaged cognitive processing.

Research Question (RQ). To what extent is a candidate EEG-derived Transcendence Index (TI), interpreted conservatively as a spectral indicator of internally oriented attention, associated with individual differences in EEG oscillatory profiles across Prayer and Relaxation?

The broader axiological concepts discussed below are presented as conceptual context; the present data support only EEG–psychological associations and Prayer–Relaxation condition differences, not direct moral, prosocial, or societal outcomes.

## 2. Theoretical Framework

### 2.1. Universal Values in Psychology and the Social Sciences

Universal values can be defined as fundamental axiological structures that express general ideals and principles guiding human behavior beyond specific cultural or historical frameworks. In psychological terms, values operate as relatively stable cognitive–motivational representations that orient individuals’ choices, evaluations, and actions in relation to the self, others, and the social environment [1,2,25]. Contemporary value science increasingly treats values as system-level representations that organize meaning, motivation, and behavior across contexts, thereby contributing to identity coherence and self-regulation [1,26].

A major reference model is Schwartz’s circumplex framework, which posits that values form a circular motivational continuum structured by compatibilities and conflicts among value priorities [3,4]. In this model, universalism and benevolence occupy the “self-transcendence” pole, reflecting concern for others’ welfare, tolerance, and social justice—orientations conceptually consistent with movement beyond egocentric interests. Empirical work indicates that value–behavior relations are systematic (though heterogeneous across value types) and preserve a coherent structure across recurrent behavioral domains [27], while cross-cultural evidence highlights that value expression depends on situational affordances and cultural context [28].

From existential and humanistic perspectives, values are also treated as core sources of meaning. Frankl’s logotherapy emphasizes value commitment as a pathway to meaning-making under adversity [9,11,29], while humanistic approaches describe “being-values” (e.g., truth, justice, unity, beauty) as markers of advanced personal development and self-transcendence beyond self-actualization [10]. Taken together, these perspectives converge on the view that values function as deep motivational organizers of identity and action, with cognitive, emotional, and behavioral manifestations [2,5,30].

### 2.2. Self-Transcendence as a Psychological Dimension

In the scholarly literature, self-transcendence is commonly defined as an individual tendency to move beyond the boundaries of the immediate self and to orient toward realities, goals, and values that surpass personal interests and egocentric concerns [10,31]. In psychobiological personality theory, Cloninger conceptualizes self-transcendence as a character dimension associated with unity, altruism, and integration of the self into a broader existential context, framing it as a non-pathological dimension of personality development [12]. Across traditions, self-transcendence is frequently linked to meaning-making and value internalization, with universal values functioning as operational expressions of transcendence under conditions of uncertainty or moral tension [9,11,32,33]. In Schwartz’s framework, self-transcendence values (universalism, benevolence) form a systematic motivational pole opposed to self-enhancement [3,4], and moral psychology suggests that value commitments shape moral intuitions and ethical evaluations in everyday decision-making [5].

From a neurocognitive viewpoint, transcendence-relevant processes (self-reflection, autobiographical meaning-making, valuation, and moral appraisal) overlap with large-scale systems supporting internal mentation, particularly the default mode network (DMN) and its interactions with executive control networks [14,15,18,34,35]. These links are consistent with valuation-based accounts emphasizing medial prefrontal involvement in self-related appraisal and meaning attribution [17,18], as well as with evidence that self-transcendent orientations can shape neural responses to persuasive information and downstream behavior [13]. In addition, experiences characterized by reduced self-focus (e.g., awe) have been associated with reduced DMN activity, supporting the conceptual bridge between transcendence and self-decentering [16].

Given the time-sensitive nature of self-reflection and valuation, EEG provides a methodologically appropriate window into transcendence-relevant neurodynamics due to its high temporal resolution and non-invasive nature [19]. Theta and alpha oscillations are consistently associated with internally oriented attention, self-monitoring, and contemplative states [20,21,36], and theta-linked mechanisms have been discussed in relation to self-transcendent experience and behavioral regulation [22]. Complementary work connects alpha/theta dynamics to mind-wandering and internally generated mentation [37,38,39] and links frontal theta dynamics to DMN-related resting-state activity [40]. Importantly for the present design, comparative findings indicate that prayer and meditation/relaxation can yield distinct alpha/theta modulation, suggesting that spiritually oriented internal focus may differ neurophysiologically from secular relaxation [23].

Finally, operationalizing a theory-informed index from EEG aligns with digital psychometrics and digital phenotyping approaches that integrate physiological signals to complement self-report and improve measurement objectivity and ecological relevance [24,29,41]. In this framework, the Transcendence Index (TI) is positioned as a candidate EEG-derived proxy of transcendence-relevant internal orientation to be interpreted cautiously pending convergent validation with established psychological measures.

### 2.3. The Transcendence Index (TI)

#### 2.3.1. EEG Spectral Features: Definition and Computation

For each participant and each experimental condition (Prayer, Relaxation), oscillatory activity was quantified by estimating spectral power within predefined frequency intervals corresponding to canonical EEG bands. Spectral power was obtained from the power spectral density (PSD), a frequency-domain representation commonly used to characterize both state-dependent changes (e.g., task/condition differences) and trait-like individual differences in neural dynamics, particularly in paradigms involving internally oriented attention and contemplative states [19,20,21]. Because absolute EEG power can vary across individuals due to non-neural factors (e.g., skull conductivity, electrode–skin impedance, scalp anatomy, baseline arousal), direct comparisons of raw power may be misleading; therefore, a normalization strategy is typically recommended to improve comparability across participants and conditions [19,21,23].

Band-limited power was extracted from the PSD within standard ranges: delta (1–4 Hz), theta (4–7 Hz), low alpha (8–10 Hz), high alpha (10–12 Hz), low beta (13–20 Hz), high beta (20–30 Hz), low gamma (30–45 Hz), and high gamma (45–70 Hz). Gamma estimates were treated with caution due to higher susceptibility to non-neural contamination (e.g., facial/neck EMG and micro-movements), and high-gamma power was retained only when recording quality permitted reliable estimation [19,42]. Because EEG power distributions are often right-skewed, logarithmic transformation is commonly applied to stabilize variance and reduce the influence of extreme values prior to parametric testing [19,20].

To emphasize frequency allocation rather than global amplitude, we computed relative band power, defined as the proportion of power in a given band relative to the total power summed across the analyzed bands. Relative power improves comparability across individuals and conditions by reducing confounding variance attributable to non-neural scaling factors and is widely used in contemplative and resting-state EEG research [20,36,42]. Relative power for band *i* was computed as

*RP_i_* = *P_i_*/Σ{*i* = 1}^{*n*}*Pi*^

where *P_i_* denotes band power in band *i* and the denominator represents total spectral power across the *n* included bands.

#### 2.3.2. Definition of the EEG–TI (Theta + Alpha Relative to Beta)

The EEG Transcendence Index (EEG–TI) was operationalized as a ratio-type spectral composite designed to quantify the balance between an internally oriented, reflective–integrative mode of neural processing and an externally oriented, task-activated mode. For each participant and condition (Prayer, Relaxation), band power was extracted for theta and alpha sub-bands (low/high alpha), and beta power was computed by combining low and high beta components to form a single reference denominator.

The numerator combines theta and alpha because these bands are treated as functionally complementary components of internal orientation. Theta activity is frequently interpreted as reflecting internal attention, monitoring, and reflective control when cognition is sustained on internally generated representations rather than incoming sensory demands. Alpha activity is commonly associated with functional inhibition of sensory input and stabilization of internally oriented processing; alpha tends to increase during eyes-closed rest and during many contemplative or reflective states, consistent with reduced external sampling and increased internal mentation [20,21,36,42]. Importantly, theta and alpha were summed not because they reflect identical processes, but because their aggregation was intended to represent a broader internal reflective–integrative mode rather than a single isolated oscillatory mechanism.

We acknowledge that alternative ratios such as theta/beta or alpha/beta may also capture meaningful aspects of internally oriented cognition. However, the present formulation was selected to represent the joint contribution of reflective monitoring (theta) and sensory gating/internal stabilization (alpha) relative to a more externally engaged beta profile. Accordingly, TI should be interpreted as a theory-informed spectral composite rather than as the only possible operationalization of transcendence-relevant neural dynamics.

Beta activity in the denominator is treated as a reference for externally oriented activation and cognitive engagement. Beta power is often elevated under conditions involving sustained alertness, analytic processing, and effortful control, and can index an “instrumental” operating mode organized around external demands and goal-directed execution. Under this interpretation, higher EEG–TI values indicate that, within the analyzed frequency content, a larger share of oscillatory energy is allocated to theta–alpha dynamics relative to beta dynamics.

Given the within-subject design contrasting Prayer and Relaxation, the most defensible use of TI is its intra-individual contrast across conditions (i.e., each participant serves as their own baseline). This reduces stable between-person scaling factors that affect absolute power and emphasizes relative spectral balance rather than raw amplitude, thereby isolating condition-specific modulation beyond a secular internal-focus control.

#### 2.3.3. Definition of the Extended EEG–TI Including Delta

In addition to the primary EEG–TI marker, we defined a delta-augmented variant by incorporating delta-band activity into the numerator. The motivation was to capture slower components of internally oriented processing that may not be fully expressed in theta–alpha dynamics alone. However, delta power requires methodological caution because it is sensitive to nonspecific factors such as vigilance fluctuations, drowsiness, and low-frequency artifacts, especially in eyes-closed paradigms. For this reason, the delta-inclusive index is treated as a secondary exploratory variant rather than the primary operationalization of transcendence-relevant dynamics. Future validation should pair delta-inclusive variants with explicit vigilance indicators (e.g., brief sleepiness ratings, behavioral proxies) and conservative low-frequency artifact handling.

#### 2.3.4. Neuroscientific Foundations of Moral Value Processing

Cognitive neuroscience converges on the view that value-related and moral processing emerges from coordinated interactions between affective valuation and cognitive control systems rather than a single “moral module.” Evidence from moral dilemma paradigms indicates reliable involvement of prefrontal circuitry, conflict-monitoring mechanisms, and limbic systems supporting affective salience and empathic responding [7]. At the network level, value-based reflection recruits systems central to self-related cognition, including the DMN for internally oriented mentation and autobiographical meaning integration, and fronto-parietal control networks supporting deliberation and norm-based evaluation under uncertainty [14,15,17,18]. EEG provides a complementary temporal perspective: theta–alpha dynamics are linked to internally oriented attention, reflective monitoring, and sensory gating, whereas higher-frequency activity (especially beta, and in some contexts gamma) can relate to cognitive engagement and integrative processing depending on task demands [8,19,20,21]. Overall, this literature supports the premise that value-laden reflection can be approached as a measurable neurocognitive phenomenon, while emphasizing the need for cautious interpretation when mapping oscillatory profiles onto complex psychological constructs.

### 2.4. Research Hypotheses and Objectives

#### 2.4.1. Hypotheses

The present study tests the overarching assumption that TI, conceptualized as a candidate EEG-derived proxy of transcendence-relevant internal orientation, is systematically associated with EEG oscillatory patterns implicated in internally oriented attention, reflective monitoring, and value-laden cognition [14,21]. Four hypotheses were specified. First, TI was expected to be positively associated with theta-band activity, consistent with the role of theta dynamics in internal attention and reflective control [20]. Second, higher TI was expected to be associated with increased alpha power, reflecting reduced external sensory sampling and stabilization of inward-focused processing [21]. Third, TI was expected to reflect a broader oscillatory configuration across bands rather than a single-band marker. Fourth, higher-frequency activity (beta/gamma) was expected to contribute to the broader oscillatory profile associated with TI; however, because TI is a relative spectral-balance composite, higher-frequency contributions were not assumed to manifest as a simple positive association [7].

#### 2.4.2. Objectives

In addition to confirmatory hypotheses, the study pursued exploratory objectives: (i) to examine whether multivariate EEG feature sets could support exploratory estimation models of TI at the individual level as part of a preliminary methodological evaluation (without claims of behavioral predictive validity), and (ii) to explore associations between TI and individual differences in personality traits (Big Five) and anxiety to provide preliminary nomological context.

## 3. Materials and Methods

### 3.1. Research Design

This study employed a quantitative, psychophysiological design centered on electroencephalography (EEG) to examine the relationship between oscillatory brain dynamics and an individual’s orientation toward universal values, operationalized via the Transcendence Index (TI). EEG was recorded under two internally oriented experimental conditions (Prayer and Relaxation), and spectral features were extracted to characterize band-limited oscillatory activity and to compute the EEG-derived TI described in Section 2.3. In parallel, participants completed a standardized psychological assessment battery (CAS++) to estimate Big Five personality traits and anxiety, allowing the study to explore whether internally oriented attention neurophysiological profiles are embedded within broader dispositional and affective differences.

In the analytic framework, TI served as the primary outcome variable derived from EEG spectral characteristics, while the predictors comprised (a) band-limited EEG power features (delta, theta, alpha, beta, gamma), (b) the spatial distribution of activity across recorded scalp locations (used as a proxy for cortical topography given the EEG configuration), and (c) individual-difference measures from CAS++ (anxiety and the five personality dimensions). This structure supports both condition-level comparisons and between-person modeling: condition contrasts (Prayer vs. Relaxation) capture within-subject modulation of oscillatory balance, whereas individual differences in TI and spectral features enable correlational and predictive analyses linking neurophysiology with personality and anxiety.

After EEG setup and impedance checking, participants completed two eyes-closed experimental blocks: Prayer and Relaxation, administered in the same session in a quiet room with minimal external stimulation. Each block lasted approximately 5 min, and blocks were separated by a brief resting interval of 1 min to reduce carryover and allow participants to reset comfortably. The order of the two conditions was counterbalanced across participants to reduce potential order and carryover effects, and this information is reported here to support protocol reproducibility. For the Prayer condition, participants were instructed to engage in their usual form of silent prayer or spiritually oriented reflection while keeping their eyes closed. They were asked to remain still, avoid speaking, and minimize facial/jaw tension to reduce electromyographic contamination. For the Relaxation condition, participants were instructed to remain quietly relaxed with eyes closed, allowing thoughts to pass naturally, without intentionally praying and without using structured contemplative techniques. The same movement-minimization instructions were applied in both blocks.

No post-condition subjective ratings or manipulation-check questionnaires were collected. The experimental conditions were therefore defined operationally by the task instructions and experimental protocol.

Because the study did not include systematic phenomenological/manipulation-check measures (e.g., perceived engagement, absorption, spirituality intensity, mind-wandering) or detailed assessment of participants’ religious background and prayer familiarity, we cannot directly determine which subjective processes differed between Prayer and Relaxation; this is acknowledged as a limitation and a priority for future research.

### 3.2. Participants

A convenience sample of 39 adults (both sexes), aged 18 to 35 years, was recruited from heterogeneous educational and socio-professional backgrounds. Eligibility criteria were set to minimize confounds that could distort EEG recordings or bias value-related cognition. Inclusion criteria consisted of (a) no known neurological or psychiatric diagnoses, (b) adequate ability to understand and follow the experimental instructions, and (c) provision of written informed consent. Exclusion criteria included (a) recent consumption of psychoactive substances prior to testing, (b) medical conditions known to affect EEG activity (e.g., epilepsy), and (c) current use of medication with central nervous system effects.

Although the sample size is relatively modest for correlational analyses involving multiple EEG frequency bands, the study was designed as exploratory research. The goal was not to establish definitive neurophysiological markers but to generate preliminary evidence and test the feasibility of the proposed spectral index under controlled experimental conditions. Such exploratory designs are common in early-stage EEG investigations that aim to identify candidate physiological patterns before larger confirmatory studies.

### 3.3. Ethical Considerations

All procedures were conducted in accordance with the ethical principles for research involving human participants, including the Declaration of Helsinki and applicable national regulations. Participants were informed about the aims of the study, the recording procedures, potential discomforts (e.g., mild fatigue during eyes-closed blocks), data confidentiality and anonymization practices, and their right to withdraw at any time without negative consequences. Written informed consent was obtained prior to participation, and study documentation and reporting followed relevant professional ethical standards [43].

### 3.4. Assessment Instruments

The Transcendence Index (TI) was defined as a composite, EEG-derived spectral index intended to approximate an individual’s tendency toward internally oriented, reflective processing. In the present study, TI was treated as a theory-informed neurophysiological proxy rather than as a directly validated psychological measure of transcendence, because no established self-transcendence scale was included in the assessment battery.

By relying on EEG-derived features rather than self-report items, TI is positioned as a complementary proxy that may reduce some declarative-report biases; however, it does not replace validated psychometric measures and requires convergent validation [24].

EEG was recorded using a Unicorn Hybrid Black (g.tec medical engineering, Vienna, Austria) wireless 8-channel system with 24-bit resolution, sampled at 250 Hz per channel [44,45,46,47,48,49,50,51]. Electrodes were positioned according to the international 10–20 system at Fz, C3, Cz, C4, Pz, PO7, Oz, and PO8, following the standard Unicorn Hybrid Black montage. The system’s reference and ground electrodes are placed on the mastoid sites, consistent with the device design. Prior to each block, electrode contact quality was checked in the acquisition software and recordings proceeded only when signal quality indicators met the manufacturer’s recommendations (the Unicorn Hybrid Black amplifier has very high input impedance, >1 GΩ).

Raw EEG streams were filtered to reduce slow drifts and line noise using a band-pass filter in the 0.5–70 Hz range and a 50 Hz notch filter (Romanian mains frequency), consistent with manufacturer guidance for obtaining clear EEG signals with this headset. Artifact handling was performed conservatively given the susceptibility of portable EEG to motion and EMG: we visually screened the recordings and excluded segments with gross artifacts (movement bursts, sustained muscle tension, or abrupt amplitude jumps) prior to spectral estimation. Only artifact-clean segments were retained for power spectral density computation; high-frequency estimates (especially gamma) were interpreted cautiously due to known EMG sensitivity in portable systems.

Power spectral density (PSD) was computed from the preprocessed EEG using FFT-based estimation on artifact-clean data, and band-limited power was derived for delta (1–4 Hz), theta (4–7 Hz), low alpha (8–10 Hz), high alpha (10–12 Hz), low beta (13–20 Hz), high beta (20–30 Hz), low gamma (30–45 Hz), and high gamma (45–70 Hz) when signal quality permitted reliable estimation. To improve comparability across participants, band-power features were summarized at the condition level (Prayer vs. Relaxation) and used to compute TI variants as described in Section 2.3. It should be noted that EEG spectral activity cannot directly identify large-scale networks such as the Default Mode Network (DMN). Therefore, interpretations linking oscillatory activity to internally oriented processing are conceptual rather than network-specific.

For spectral estimation, the continuous EEG signal was segmented into non-overlapping epochs of approximately 2 s. Power spectral density was estimated using Fast Fourier Transform (FFT) with a Hanning window applied to each epoch in order to reduce spectral leakage and stabilize frequency estimation. The frequency resolution was determined by the epoch duration and the device sampling rate. Artifact rejection procedures were applied prior to spectral estimation to remove segments contaminated by eye blinks, abrupt movement, or sustained muscle activity. Segments exceeding predefined amplitude thresholds or showing clear EMG contamination were excluded following visual inspection. Across participants, approximately 8–12% of recorded epochs were excluded during artifact screening, with the remaining clean segments retained for spectral power computation across the predefined frequency bands.

Recordings were conducted in a controlled environment with minimal external stimulation. Participants completed two eyes-closed blocks corresponding to the experimental conditions (Prayer and Relaxation), chosen to contrast spiritually oriented internal focus with a non-spiritual internal-focus control while keeping sensory input and overt behavior as constant as possible.

Personality traits were assessed using a brief instrument derived from the International Personality Item Pool (IPIP), an open scientific resource developed for the measurement and validation of personality constructs (International Personality Item Pool: A Scientific Collaboratory for the Development of Advanced Measures of Personality Traits and Other Individual Differences, available at http://ipip.ori.org/). The instrument measures the five major personality dimensions commonly referred to as the Big Five: Extraversion, Agreeableness, Conscientiousness, Emotional Stability, and Autonomy. Participants responded to the items using a 5-point Likert scale ranging from 1 (strongly disagree) to 5 (strongly agree). Separate scores were calculated for each personality dimension. Extraversion reflects sociability, energetic engagement, and positive affect; Agreeableness captures interpersonal warmth, empathy, and cooperative orientation; Conscientiousness represents organization, persistence, and goal-directed behavior; Emotional Stability refers to resilience to stress and effective emotional regulation; and Autonomy reflects independence in judgment, self-directed decision making, and personal agency. These personality dimensions were included in the analysis in order to explore potential associations between stable dispositional traits and the EEG-derived transcendence index proposed in the present study. Although these measures offered a broader dispositional context for interpreting TI-related individual differences, they were not designed to assess self-transcendence directly and therefore do not provide convergent construct validation for the index. Pearson correlations were used for the initial association analyses. Because multiple correlations were tested, false discovery rate (FDR) control using the Benjamini–Hochberg procedure (q = 0.05) was applied within each correlation family.

## 4. Results

Descriptive statistics were computed for anxiety, Big Five personality dimensions (CAS++), and EEG band-power features (delta, theta, alpha, beta, gamma). The analytic sample included *N* = 39 participants with complete data for the main psychological variables and principal EEG band features (with one missing value for high alpha; see Table 1 and Table 2). As shown in Table 1, personality scores were generally centered in the upper-mid range of the scales, whereas EEG spectral power features showed substantial between-participant variability, particularly for delta and theta power. Distributional indices further indicated pronounced positive skewness and kurtosis for low-frequency power (especially delta and theta), consistent with the typical right-skewed nature of raw spectral power values in EEG datasets.

On the psychological side, mean anxiety was moderate (M = 5.59, SD = 4.96), and Big Five-related dimensions showed relatively high central tendency, especially agreeableness (M = 79.05, SD = 9.08). On the neurophysiological side, absolute band-power values displayed wide ranges, with the largest dispersion observed in delta power (SD ≈ 6283) and theta power (SD ≈ 1739). The strong positive skewness and elevated kurtosis for delta and theta (as indicated in the SPSS v26 output) suggest that subsequent inferential analyses involving raw power values should account for non-normality (e.g., via log-transformed features and/or ratio-based indices such as TI) to reduce sensitivity to extreme observations and improve interpretability.

Table 2 reports descriptive statistics for the sub-band spectral features (low/high alpha; low/high beta; low/middle gamma) and for the two EEG-derived transcendence indices (TI_EEG and the delta-augmented variant TI_EEG_D). The available sample size was *N* = 39 for all variables, except high alpha, which had *N* = 38 due to one missing value. Consistent with typical EEG power distributions, several sub-band power variables—most notably low alpha and the gamma sub-bands—showed substantial dispersion and positive skew, whereas the TI indices displayed comparatively compact ranges, reflecting their ratio-based construction.

TI_EEG_D was included as an exploratory delta-augmented sensitivity variant rather than as a systematic ablation analysis.

Sub-band power estimates exhibited marked interindividual variability, particularly in low alpha (M = 5469.42, SD = 3156.72), consistent with alpha’s sensitivity to internal attentional state and eyes-closed conditions. Beta sub-bands were comparatively less dispersed, while gamma sub-bands showed wide ranges, which is expected given the greater susceptibility of higher-frequency power to non-neural influences (e.g., subtle muscle activity) and signal-to-noise constraints. In contrast, both TI indices showed relatively constrained variability (TI_EEG range: 0.47–2.44; TI_EEG_D range: 0.78–2.76), supporting their use as stabilized summary markers for subsequent association and group/condition analyses.

To directly examine whether the two internal-focus conditions produced distinguishable spectral profiles, within-subject comparisons were conducted between Prayer and Relaxation states. A paired-samples *t*-test indicated that TI values were higher during Prayer than during Relaxation, t(38) = 2.18, *p* = 0.035, corresponding to a small-to-moderate effect size (Cohen’s d = 0.35, 95% CI [0.03, 0.67]). Given the exploratory design and modest sample size (*N* = 39), these findings should be interpreted cautiously as preliminary evidence that the two internal-focus conditions produce partially distinguishable spectral dynamics.

Table 3 presents Pearson correlations among the EEG band/sub-band power features and the two EEG-derived transcendence indices (TI_EEG and TI_EEG_D). Overall, the spectral bands showed the expected interdependence (e.g., delta with theta; alpha sub-bands with beta sub-bands), while the TI indices exhibited a clear pattern of associations consistent with their construction. Specifically, TI_EEG was positively related to theta and low alpha, and negatively related to high beta, whereas TI_EEG_D showed stronger positive associations with delta in addition to theta and a stronger negative association with beta components. The two indices were strongly correlated with each other, indicating substantial shared variance while still preserving distinct contributions from delta in the delta-augmented variant.

Because TI is algebraically constructed from theta–alpha relative to beta activity, correlations between TI and its component bands are mathematically expected and are reported for transparency rather than interpreted as evidence of construct validity.

The strongest positive associations were observed among adjacent low-frequency components (delta–theta, theta–low alpha), reflecting shared variance in slow oscillatory activity. As expected for an index emphasizing internally oriented dynamics relative to beta activity, TI_EEG correlated positively with theta (r = 0.547, *p* < 0.001) and low alpha (r = 0.592, *p* < 0.001), and negatively with high beta (r = −0.592, *p* < 0.001). The delta-augmented index (TI_EEG_D) showed an additional strong positive relationship with delta (r = 0.586, *p* < 0.001) and a moderate negative relationship with low beta (r = −0.343, *p* = 0.032), consistent with the fact that delta contributes directly to this variant. Finally, TI_EEG and TI_EEG_D were strongly intercorrelated (r = 0.811, *p* < 0.001), indicating that both indices capture a common underlying oscillatory balance while TI_EEG_D additionally reflects low-frequency (delta) variance.

Table 4 summarizes Pearson correlations between the two EEG-derived transcendence indices (TI_EEG and TI_EEG_D), anxiety, Big Five-related dimensions (CAS++: Autonomy, Extraversion, Agreeableness, Conscientiousness, Emotional Stability), and the principal EEG band features (Alpha, Beta, Theta). Overall, the pattern indicates that the TI indices were systematically associated with both neurophysiological dynamics and selected psychological variables, offering preliminary nomological context rather than direct construct validation.

All analyses reported below are correlational and exploratory; no behavioral tasks or prospective outcomes were included, and therefore no claims about behavioral prediction are made. Where applicable, we highlight associations that remained significant after FDR correction, while treating nominal-only effects as exploratory trends.

Anxiety showed a strong negative association with emotional stability (r = −0.593, *p* < 0.001), consistent with construct expectations. The delta-augmented transcendence index (TI_EEG_D) was positively related to anxiety (r = 0.469, *p* = 0.003), whereas the simpler index (TI_EEG) showed only a nonsignificant positive trend (r = 0.293, *p* = 0.070). Regarding personality, both TI indices were negatively associated with extraversion (TI_EEG: r = −0.335, *p* = 0.037) and agreeableness (TI_EEG: r = −0.330, *p* = 0.040), while emotional stability displayed small-to-moderate negative associations with both TI_EEG (r = −0.363, *p* = 0.023) and TI_EEG_D (r = −0.368, *p* = 0.021). These patterns suggest that higher EEG-derived transcendence scores co-occurred with lower sociability/positive interpersonal orientation in this sample and with greater emotional lability, particularly when delta was included.

Unless stated otherwise, interpretation prioritizes associations that remain significant after FDR correction; nominal-only effects are treated as exploratory trends.

As expected from their spectral construction, TIs were strongly tied to EEG band dynamics. TI_EEG was positively associated with alpha (r = 0.480, *p* = 0.002) and theta (r = 0.547, *p* < 0.001), and it was negatively associated with beta (r = −0.317, *p* = 0.050). TI_EEG_D showed an even stronger negative association with beta (r = −0.437, *p* = 0.005) and a strong positive association with theta (r = 0.558, *p* < 0.001). Finally, the canonical coupling among EEG bands was also observed (alpha–theta: r = 0.598, *p* < 0.001; alpha–beta: r = 0.487, *p* = 0.002; beta–theta: r = 0.346, *p* = 0.031), supporting internal consistency of the spectral feature extraction.

## 5. Discussion

### 5.1. Interpreting the Findings in Relation to the Hypotheses

Before interpreting the psychological implications of the findings, an important methodological caveat must be acknowledged. The Transcendence Index (TI) is algebraically derived from combinations of EEG spectral components, specifically the relative contribution of theta–alpha activity compared to beta power. Consequently, correlations between TI and individual frequency bands should not be interpreted as independent empirical relationships but rather as mathematical properties of the index construction itself.

In light of this constraint, the present findings should not be interpreted as direct neural evidence of the psychological construct of transcendence. Instead, the Transcendence Index is treated more conservatively as a candidate spectral indicator of internally oriented attention, which may conceptually overlap with theoretical descriptions of transcendence in psychological literature. Importantly, the present study does not provide direct evidence of Default Mode Network (DMN) activation. The spatial resolution of scalp EEG does not allow the reliable identification of large-scale functional networks; therefore, references to the DMN are used only as a conceptual framework for understanding internally oriented attentional processes described in the cognitive neuroscience literature. Accordingly, the observed oscillatory patterns should be interpreted as spectral correlates of internally oriented attention rather than as direct markers of DMN activity.

Finally, the findings must be considered within the exploratory nature of the study. Given the modest sample size (*N* = 39), the results cannot be interpreted as definitive evidence of stable neurophysiological associations. Rather, they provide preliminary support for the feasibility of the proposed candidate index and serve as a starting point for future studies with larger samples and more advanced statistical modeling.

Overall, the findings support the more cautious premise that the proposed TI—interpreted as a candidate EEG-derived proxy of internally oriented, reflective processing—shows systematic associations with scalp EEG oscillatory patterns in an eyes-closed internal-focus paradigm. TI showed robust associations with theta-band dynamics, aligning with evidence that theta activity indexes internally oriented attention, reflective monitoring, and control processes that become prominent in introspective states [20,21], reflecting the expected mathematical behavior of the index given its algebraic construction. Importantly, this pattern emerged despite the use of a portable low-density EEG system, suggesting that the TI captures a relatively stable oscillatory balance rather than nonspecific individual differences in absolute amplitude [19].

TI was positively related to alpha activity, a finding consistent with the functional interpretation of alpha as reflecting sensory gating and stabilization of internal mentation, particularly in eyes-closed and inward-focused contexts [20,21], reflecting the expected mathematical behavior of the Transcendence Index (TI), which is derived from theta–alpha relative to beta activity. Within this framework, higher TI values can be interpreted as reflecting a more pronounced shift toward internal monitoring and reduced dependence on external stimulation. The observed coupling between theta and alpha also fits the view that internally oriented attention is supported by coordinated oscillatory processes that jointly enable reflective attention and integrative monitoring, rather than by a single isolated rhythm.

Consistent with H3, the correlation structure suggests that internally oriented attention is best characterized as a neurodynamic profile spanning multiple frequency components. Accordingly, the study treated beta/gamma activity as part of the overall spectral architecture of TI, not as stand-alone markers expected to increase independently of the theta–alpha profile. Such an interpretation is theoretically coherent with network-level accounts of meaning-making and self-referential cognition that emphasize internally directed processing and sustained reflective control [14]. This pattern is consistent with the logic of the index construction, in which theta and alpha were combined to reflect complementary dimensions of internal orientation, namely reflective monitoring and sensory gating, contrasted against beta as a proxy of externally engaged activation.

Finally, the negative association with beta activity again reflects the mathematical structure of the index rather than an independent empirical finding. Although the initial hypothesis assumed that higher-frequency activity could be relevant to the cognitive-regulatory aspects of internally oriented attention processing, the present findings did not support a simple positive association between TI and beta activity. Instead, the data suggest that higher TI values were characterized by a spectral profile in which theta–alpha dynamics were relatively more prominent than beta activity. This pattern is better interpreted as reflecting the predominance of internally oriented reflective processing over externally engaged analytic activation, rather than as evidence that higher-frequency control-related activity increases together with TI. We have revised the interpretation accordingly in order to align the discussion more closely with the observed correlation structure.

In other words, the present results support a profile-based interpretation of TI, in which internally oriented attention is expressed through the relative balance among oscillatory components, not through uniformly elevated activity across all cognitively relevant frequency bands.

Although beta and gamma rhythms are frequently implicated in cognitive integration, the present operationalization treats them as indices of task-activation and executive engagement that counterbalance theta–alpha internal orientation. From a moral neuroscience perspective, normative evaluation and complex moral decision-making recruit prefrontal control systems interacting with affective valuation processes [7], and oscillatory markers in the higher-frequency range may reflect this control-demand component. At the same time, the present correlational findings indicate that the TI is primarily driven by the reflective/internal axis (theta–alpha relative to beta), suggesting that transcendence in this sample may be more strongly expressed through internal monitoring and sensory gating than through heightened task-analytic activation.

Importantly, the present data do not support a normative interpretation of higher TI as inherently adaptive or prosocial. The observed associations (e.g., the delta-augmented TI_EEG_D with higher anxiety and lower emotional stability; and TI_EEG with lower extraversion) suggest that the index may capture a broader internal-orientation or vigilance-related profile that can accompany both reflective meaning-making and anxiety-linked internal monitoring. Future work should therefore test whether TI shows incremental validity for specifically self-transcendent outcomes (e.g., self-transcendence scales, value-based choices, prosocial behavior) beyond personality traits.

Importantly, the empirically independent findings of the study concern the spectral differences observed between the Prayer and Relaxation conditions, as well as the associations between the Transcendence Index and personality traits. Because these results do not derive from the algebraic construction of the index, they represent the main substantive empirical contributions of the present study.

### 5.2. Theoretical Contributions to Understanding Transcendence and Values

The study advances the exploration of internally oriented attention as a psychologically meaningful domain that may be approached through measurable neurophysiological patterns. By operationalizing TI using EEG-derived spectral features, the present approach moves beyond exclusively self-report-based value assessment and proposes an integrative framework that connects value psychology with existential perspectives and cognitive neuroscience. In line with contemporary calls to enrich psychological measurement with objective behavioral and physiological signals, an EEG-derived TI can be viewed as a step toward digital psychometrics, where latent dispositions are inferred from reproducible neurocognitive features rather than solely from declarative responses [24]. At the level of mechanism, the relevance of frontal midline theta is theoretically meaningful: it differentiates separable cognitive control strategies while still generalizing the broader need for cognitive control, offering a plausible bridge between reflective monitoring and adaptive regulation in value-related states [52].

References to large-scale systems (e.g., DMN) and moral-value circuitry are provided as theoretical context and should not be interpreted as direct empirical inferences from the present low-density scalp EEG data.

At the same time, the present findings should not be interpreted as establishing construct validity for TI as a measure of transcendence in the strict psychometric sense. Because the study did not include a validated self-transcendence instrument, the current results are better understood as supporting the feasibility of a theory-informed EEG-derived proxy rather than confirming that TI directly measures the psychological construct of transcendence. Future studies should therefore examine convergent and discriminant validity by pairing TI with established self-transcendence and value-orientation scales.

Beyond oscillatory markers, the present framework can be situated within emerging perspectives that emphasize temporality as a defining property of advanced self-transcendent experience. Recent work on non-duality in expert meditators highlights the potential role of intrinsic neural time-scales in shaping experience, suggesting that transcendence may involve not only which networks activate, but also how neural dynamics unfold and stabilize across time [53].

More broadly, the present findings are consistent with the possibility that value-laden reflection may be accompanied by measurable oscillatory profiles; however, the current data do not allow direct inferences about the neural grounding of universal values or specific large-scale network mechanisms. In this sense, transcendence can be framed as an emergent neuropsychological configuration that integrates (i) internally oriented attention and reflective monitoring, (ii) the maintenance of self-referential meaning structures that support coherence over time, and (iii) regulatory balance relative to externally oriented activation. This interpretation is consistent with evidence that internally directed cognition and autobiographical integration are supported by large-scale systems such as the DMN [14], which provide a plausible theoretical framework for interpreting internally oriented mentation, but they are not empirically tested in the present low-density EEG design. Importantly, self-processing accounts also show that DMN dynamics interact with embodied and social-affective mechanisms (including links to the mirror neuron system), supporting the idea that value integration can remain simultaneously introspective and socially grounded [54].

Methodologically, the present approach is also compatible with a broader measurement logic in which psychological constructs are treated as information-bearing patterns. From an information-theoretic viewpoint, meaningful psychological signals can be described through variability, structure, and communication-like properties of data streams, an interpretive frame that aligns well with EEG-derived indices when carefully operationalized and transparently reported [55]. In parallel, anchoring the TI construct in experimental logic (clear task design, reproducibility, and interpretability of psychophysiological markers) reinforces the scientific legitimacy of the operationalization, especially when the inferential claims remain proportionate to the available evidence [56,57,58].

At the level of neurodynamic interpretation, evidence from experienced meditators further supports the functional meaning of theta–alpha configurations. Alpha–theta cross-frequency dynamics vary systematically across meditation, rest, and cognitively demanding tasks, suggesting that TI-related oscillatory patterns can index shifts between thoughtless awareness, reflective monitoring, and effortful cognition, exactly the kind of flexible regulation expected in value- and meaning-centered states [59]. Converging fMRI evidence also indicates that self-transcendent versus self-enhancement core values are associated with distinguishable activation patterns, strengthening the claim that value orientations can be mapped onto reproducible neural configurations rather than treated as purely declarative commitments [60]. Finally, acknowledging that contemplative and transcendent states can be embodied and sensory-mediated (not only cognitively framed) helps interpret TI as a generalizable signature across heterogeneous internal-focus practices, including embodied sonic meditation, while still preserving conceptual space for practice-specific phenomenology [61]. Future work may benefit from extending the present spectral approach using nonlinear and attention-based temporal–frequency modeling frameworks, especially when integrating EEG with systems-level neuroimaging such as fMRI. High-dimensional neuroimaging signals often exhibit nonlinear structures, incomplete sampling, and complex temporal–frequency dependencies, which can challenge conventional linear feature extraction. Recent machine-learning work has proposed methods that combine temporal–frequency attention with deep nonlinear factorization to improve representation learning and classification performance in high-dimensional neuroimaging contexts. For example, Ke and collaborators [62] illustrate how attention-guided factorization can support more refined temporal–frequency feature extraction and interpretation. Similar approaches could be adapted to EEG-based indices (including TI) to evaluate nonlinear mappings between oscillatory profiles and psychological constructs, potentially improving robustness beyond linear correlational analysis.

At the same time, the present results should not be interpreted as establishing direct construct validity for TI as a psychological measure of transcendence. Because no validated self-transcendence scale was administered, the study cannot determine the degree to which TI converges with established measures of internally oriented attention. The inclusion of personality and anxiety measures offered only a preliminary nomological context, showing how the EEG-derived index relates to broader dispositional variables, but these constructs cannot substitute for direct convergent validation. Future studies should therefore pair TI with validated self-transcendence or value-orientation scales in order to test its psychometric status more rigorously.

Because both experimental blocks were eyes-closed and internally oriented, theta–alpha predominance may partially reflect general eyes-closed resting-state physiology and sensory gating rather than transcendence-specific processing. In addition, reduced vigilance/drowsiness can increase low-frequency power, which is particularly relevant for delta-inclusive formulations (TI_EEG_D). Finally, individual differences in internal orientation may overlap with broader dispositional tendencies such as lower extraversion or higher anxiety, which could shift oscillatory balance toward slower rhythms without necessarily implying an adaptive, mature, or prosocial orientation. For these reasons, TI is best interpreted in the present study as a candidate EEG-derived proxy of internal reflective orientation under eyes-closed conditions, whose transcendence-specific meaning requires convergent validation (self-transcendence/value scales), vigilance monitoring, and behavioral criteria in future research. Because the present study used low-density scalp EEG and an exploratory correlational design, references to large-scale systems (e.g., DMN) and moral-value circuitry are presented as theoretical context rather than direct empirical inference from the current data.

A further methodological priority for future validation is component (ablation) testing of the TI formulation. Because TI is a composite ratio index, it is important to evaluate whether its associations with psychological variables are driven primarily by one spectral component (theta, alpha, or beta) or by the intended balance among them. Future studies should therefore compare reduced variants (e.g., theta/beta, alpha/beta, theta/alpha) and component-removed versions to determine the most stable and interpretable operationalization.

### 5.3. Neuropsychological and Axiological Implications

The following axiological considerations are offered as interpretive context. Given the correlational design and the absence of behavioral outcomes, they should not be interpreted as direct empirical implications of TI for moral responsibility, prosocial behavior, or societal cohesion.

At a neuropsychological level, the results suggest that orientation toward universal values is supported by coordinated activity patterns consistent with the interplay between systems involved in self-referential processing and reflective control. The prominence of theta–alpha dynamics in relation to TI is consistent with models in which introspection, internal monitoring, and attentional gating form the neurocognitive foundation for meaning-making and value integration [20,21]. At the same time, the observed relationships with beta dynamics underscore that internally oriented attention is not purely contemplative or affective; it is embedded within broader regulatory architectures that balance internal reflection with externally oriented activation and goal-directed engagement.

From an axiological perspective, the present findings are consistent with the idea that value-laden reflection and meaning-oriented cognition may have measurable neurophysiological correlates. However, the current results do not permit normative conclusions (e.g., moral responsibility or prosociality) because no behavioral outcomes were assessed. Accordingly, the axiological interpretation is kept at the level of conceptual alignment with models linking internal reflection, self-referential processing, and value orientation. These implications are consistent with the idea that moral judgment depends on coordinated interactions between affective valuation and executive control systems [7], and that individual differences in these interactions can be detected in electrophysiological dynamics [8].

### 5.4. Comparison with Prior Research

The present results align with prior work highlighting the relevance of theta and alpha oscillations for introspection, meditation-related states, and self-referential processing [20,21]. The broader framing is also compatible with system-level accounts emphasizing internally oriented cognition supported by the DMN and related networks [14]. In addition, the theoretical relevance of higher-frequency dynamics for cognitive control and normative evaluation remains consistent with moral neuroscience evidence, even though, in the present study, beta activity entered the observed TI profile primarily as a relative contrast term rather than as a positively associated component [7].

At the same time, the current study extends this literature by integrating spectral features into a single composite marker (TI) that can be computed from portable EEG recordings and used in a digital psychometrics logic. This contribution is methodological as well as theoretical: it offers a replicable operationalization that can complement existing models of transcendence and universal values while remaining feasible in applied contexts that cannot support high-density EEG or fMRI.

### 5.5. Limitations and Future Research

A major limitation of the present study is that TI was not validated against an established self-transcendence scale or related external criterion. As a result, the construct validity of the index remains preliminary, and the current findings should be interpreted as evidence for the feasibility of an EEG-derived proxy rather than as confirmation of a validated spectral correlate of internally oriented attention. An additional limitation concerns the initial formulation of the higher-frequency hypothesis. Because TI is a ratio-based composite emphasizing relative spectral balance, the role of beta/gamma activity should be interpreted within this comparative architecture rather than in terms of simple directionally positive effects. Future studies may benefit from separating absolute band effects from relative composite-index effects.

A further limitation is that the study did not include behavioral indicators typically associated with internally oriented attention, such as prosocial choices, moral judgment performance, or value-consistent decision-making tasks. As a result, the present findings cannot support claims about TI as a predictor of internally oriented attention behavior. Future research should examine criterion and predictive validity by pairing TI with behavioral paradigms (e.g., moral dilemma tasks, prosocial allocation games, value-based decision tasks) and by testing whether TI explains variance in these outcomes beyond personality and affective measures.

An additional interpretative issue concerns the pattern of personality associations observed in the present data. Higher Transcendence Index (TI) values were associated with lower extraversion, lower agreeableness, and lower emotional stability. At first glance, this pattern may appear theoretically counterintuitive if transcendence is conceptualized as an inherently prosocial or emotionally integrated psychological state. However, when the TI is interpreted more conservatively as reflecting internally oriented attentional engagement rather than transcendence itself, the pattern becomes more understandable. Individuals lower in extraversion may naturally engage more frequently in introspective or inwardly focused cognitive states, while reduced emotional stability may be associated with heightened internal monitoring and self-referential processing. Within this framework, the observed associations may reflect differences in dispositional attentional orientation rather than direct psychological correlates of transcendence. Given the exploratory nature of the study, these findings should therefore be interpreted cautiously as preliminary indications that the proposed EEG-derived index may capture broader individual differences in internally oriented cognitive processing. Future research combining EEG markers with validated measures of self-transcendence and contemplative experience will be necessary to clarify these relationships.

We also included a delta-augmented variant of the index (TI_EEG_D) as an exploratory sensitivity check, because slow-frequency activity may co-occur with deep internally oriented states in some contemplative contexts. However, this was not intended as a systematic ablation study. Delta power is particularly sensitive to nonspecific factors such as vigilance fluctuations, drowsiness, and low-frequency recording artifacts, especially in eyes-closed paradigms, which can complicate interpretation. Accordingly, TI_EEG_D is best treated as a secondary exploratory variant, and future work should implement a structured ablation/robustness framework comparing multiple formulations (e.g., theta/beta, alpha/beta, (theta + alpha)/beta, and delta-augmented variants) under explicit vigilance control to determine the most stable and interpretable operationalization.

A further limitation concerns circularity: because TI is computed directly from theta/alpha relative to beta, associations between TI and these spectral components are definitional and cannot be used to validate TI as a marker of transcendence. Accordingly, the present study treats these component correlations as descriptive checks of index behavior; construct validation requires external criteria and incremental validity testing beyond component bands. Another limitation concerns the interpretation of EEG oscillations in relation to large-scale brain networks. Because scalp EEG cannot directly measure network-level activity such as the Default Mode Network (DMN), any conceptual connection between the observed oscillatory patterns and DMN-related processes should be interpreted cautiously. Also, due to the lack of manipulation checks, the possibility that the participants’ actual psychological states overlapped between the two conditions cannot be ruled out. Future research should combine EEG measures with subjective experiential reports in order to better link spectral dynamics with participants’ internally oriented cognitive states.

Given the modest sample size (*N* = 39), the statistical analyses were intentionally limited to exploratory correlations (with FDR control) rather than multivariable models with multiple covariates. The relatively small sample size (*N* = 39) limits the statistical power and generalizability of the correlational analyses. The results should therefore be interpreted as preliminary exploratory findings, requiring replication in larger samples before firm conclusions can be drawn. Future studies with larger samples should test multivariate models to examine whether TI explains variance beyond anxiety, personality traits, and baseline spectral profiles, and should evaluate generalizability using cross-validation or out-of-sample prediction.

## 6. Conclusions

The primary aim of this study was to examine individuals’ orientation toward universal values using an EEG-derived Transcendence Index (TI) computed from spectral characteristics of brain activity. By integrating perspectives from value psychology, existential psychology, and cognitive neuroscience, the study advances a digital-psychometrics approach in which internally oriented attention is explored through a theory-informed EEG-derived spectral composite.

The findings indicate that TI is meaningfully related to specific EEG activity patterns, supporting the premise that internally oriented attention may be associated with identifiable neurophysiological profiles. In particular, higher TI values were associated with enhanced theta-band activity, consistent with the involvement of introspective attention, internal monitoring, and self-referential processing in internally oriented attention functioning. In parallel, TI’s positive association with alpha-band power, especially over posterior regions, suggests the contribution of internal self-regulatory mechanisms and cognitive-affective integration, consistent with an inward-focused processing mode.

Importantly, the results support a profile-based interpretation: higher transcendence was expressed through a coherent configuration combining elevated theta–alpha dynamics alongside comparatively moderated beta- and gamma-range activation. This reinforces the conclusion that transcendence cannot be reduced to a single EEG marker; rather, it represents an emergent construct arising from coordinated interactions among multiple oscillatory processes. The observed beta-related findings suggest that the TI profile is better understood as a relative oscillatory balance favoring internally oriented reflective dynamics, rather than as a generalized increase across all cognitively relevant frequency bands.

A key contribution of the study is methodological. By proposing an EEG-derived TI, the present work complements self-report approaches that can be constrained by social desirability, limited introspective access to personal value priorities, and self-evaluation distortions. EEG provides a direct window into neural dynamics associated with reflective, moral, and existential processes, including mechanisms that operate partially outside conscious awareness. In this sense, TI can be viewed as a preliminary EEG-derived proxy of internally oriented attention with potential relevance for contemporary frameworks of digital psychometrics and biomarker-informed assessment.

The use of a ratio-based index (theta + alpha relative to beta), implemented on a portable, low-density EEG system, also strengthens the applied value of the approach by supporting feasibility, transparency, and replicability across contexts where high-density neuroimaging is not available. At the same time, these findings should be interpreted within the constraints of the present design and sample size, and future work should expand validation using larger samples, stronger vigilance controls (particularly for delta-inclusive variants), and cross-method convergence with established self-report measures of values and transcendence. However, because the study did not include a validated self-transcendence measure, TI should presently be regarded as an exploratory EEG-derived proxy whose construct validity remains to be established. These findings should be interpreted in light of the limitations discussed above.

In conclusion, the present study supports the view that transcendence is a complex psychological dimension grounded in neurocognitive functioning and closely related to the orientation toward universal values. The EEG-derived Transcendence Index offers a promising exploratory methodological bridge between neuroscience, value science, and applied psychology, although its construct validity remains to be established through direct comparison with validated psychological measures of transcendence. Future studies are required to test whether TI shows criterion and predictive validity for internally oriented attention behaviors, which were not assessed in the present design. Future work should also include systematic ablation analyses to test the incremental contribution of theta, alpha, and beta components to the TI–psychological associations. Relatedly, future work should systematically compare alternative spectral formulations to test whether the observed psychological and neurophysiological associations are specific to the current TI operationalization or generalize across plausible band combinations. In addition to the present (theta + alpha)/beta formulation, comparative testing of theta/beta and alpha/beta ratios, as well as variants using separate alpha sub-bands (e.g., low vs. high alpha) and beta sub-bands, may clarify which combination yields the most stable, interpretable, and reproducible pattern across internal-focus conditions. Future methodologies should evaluate delta-inclusive variants only within systematic ablation designs and with explicit vigilance control, given the nonspecific sensitivity of delta activity in eyes-closed conditions.

## Figures and Tables

**Table 1:** Descriptive statistics for psychological measures and EEG band-power features (*N* = 39).

Variable	*N*	M	SD	Median	Min	Max
Anxiety level	39	5.59	4.96	4.00	0.00	18.00
Emotional stability	39	64.49	13.68	67.00	33.00	86.00
Autonomy	39	74.28	12.92	75.00	51.00	92.00
Extraversion	39	72.26	12.13	71.00	44.00	92.00
Agreeableness	39	79.05	9.08	80.00	56.00	94.00
Conscientiousness	39	70.41	10.05	72.00	46.00	88.00
Delta power	39	9327.87	6283.00	7842.11	3091.62	35,402.24
Theta power	39	3849.94	1739.33	3431.81	2070.69	10,319.16
Alpha power	39	4985.45	1877.78	4686.50	2388.21	10,632.81
Beta power	39	1792.57	490.66	1633.04	1160.91	2924.03
Gamma power	39	693.70	300.05	620.91	405.96	1762.24

**Table 2:** Descriptive statistics for EEG sub-band power features and EEG-derived Transcendence Indices.

Variable	*N*	M	SD	Median	Min	Max
Low alpha power	39	5469.42	3156.72	4481.29	2075.85	18,185.49
High alpha power	38	4729.73	1787.51	4334.27	1304.65	8001.07
Low beta power	39	2109.69	618.12	1910.07	963.98	3584.82
High beta power	39	1415.47	504.76	1313.15	391.09	2837.45
Low gamma power	39	943.36	413.81	847.22	250.43	2323.46
Middle gamma power	39	478.68	281.22	408.09	215.24	1623.94
TI_EEG	39	1.36	0.36	1.38	0.47	2.44
TI_EEG_D	39	1.87	0.38	1.90	0.78	2.76

Note. High alpha had one missing observation (*N* = 38). TI_EEG and TI_EEG_D are log-scaled composite indices derived from band-power features, which typically results in narrower ranges relative to raw power values.

**Table 3:** Pearson correlations among EEG power features and EEG-derived Transcendence Indices.

Variable	1	2	3	4	5	6	7	8
1. Delta	—							
2. Theta	0.670 **	—						
3. Low alpha	0.245	0.709 **	—					
4. High alpha	0.212	0.315	0.440 **	—				
5. Low beta	0.188	0.353 *	0.474 **	0.650 **	—			
6. High beta	0.067	0.104	0.134	0.225	0.663 **	—		
7. TI_EEG	0.155	0.547 **	0.592 **	0.240	−0.220	−0.592 **	—	
8. TI_EEG_D	0.586 **	0.558 **	0.365 *	0.077	−0.343 *	−0.607 **	0.811 **	—

Note. Two-tailed Pearson correlations are reported. To control for multiple comparisons, *p*-values were adjusted using the Benjamini–Hochberg false discovery rate (FDR) procedure (q = 0.05) within each correlation table. Effects are interpreted as robust only when they remain significant after FDR correction; nominal (uncorrected) significance is indicated with * (*p* < 0.05) and ** (*p* < 0.01).

**Table 4:** Pearson correlations among anxiety, personality traits, TI indices, and EEG band features (*N* = 39).

Variable	1	2	3	4	5	6	7	8	9	10	11
1. Anxiety	—										
2. TI_EEG	0.293	—									
3. TI_EEG_D	0.469 **	0.811 **	—								
4. Autonomy	−0.184	−0.038	−0.146	—							
5. Extraversion	−0.283	−0.335 *	−0.147	0.112	—						
6. Agreeableness	−0.317 *	−0.330 *	−0.300	−0.145	0.138	—					
7. Conscientiousness	−0.150	−0.220	−0.219	0.379 *	0.039	0.221	—				
8. Emotional stability	−0.593 **	−0.363 *	−0.368 *	0.495 **	0.393 *	0.304	0.603 **	—			
9. Alpha	−0.230	0.480 **	0.249	−0.007	−0.031	−0.200	−0.116	−0.029	—		
10. Beta	−0.301	−0.317 *	−0.437 **	0.240	0.063	0.078	−0.001	0.123	0.487 **	—	
11. Theta	0.233	0.547 **	0.558 **	0.165	−0.126	−0.354 *	−0.179	−0.336 *	0.598 **	0.346 *	—

Note. Two-tailed Pearson correlations are reported. To control for multiple comparisons, *p*-values were adjusted using the Benjamini–Hochberg false discovery rate (FDR) procedure (q = 0.05) within each correlation table. Effects are interpreted as robust only when they remain significant after FDR correction; nominal (uncorrected) significance is indicated with * (*p* < 0.05) and ** (*p* < 0.01).

## Data Availability

The data supporting the findings of this study are available from the first author upon reasonable request due to ethical and data protection considerations.
